# Safety and Efficacy of Anticoagulation in Patients with Cirrhosis: A Meta-Analysis

**DOI:** 10.1155/2021/8859602

**Published:** 2021-04-21

**Authors:** Huan Chen, Jiaming Lei, Sicheng Liang, Gang Luo, Mingming Deng, Muhan Lü

**Affiliations:** Department of Gastroenterology, The Affiliated Hospital of Southwest Medical University, Luzhou, Sichuan Province, China

## Abstract

**Background and Aims:**

Portal vein thrombosis is a serious adverse event that occurs during liver cirrhosis. We performed a meta-analysis to evaluate the safety and efficacy of anticoagulant therapy and prophylactic anticoagulant therapy in cirrhosis patients with (/without) portal vein thrombosis.

**Methods:**

Eligible comparative studies were identified by searching the following electronic databases: PubMed, Embase, Cochrane Library, Web of Science, and CNKI. A meta-analysis was performed to calculate odds ratios and 95% confidence intervals using fixed-effects models. Recanalization and thrombus progression were defined as the primary outcomes. Secondary outcomes included adverse events and death mortality.

**Results:**

A total of 3479 patients were included in this analysis. Compared with the control group, the recanalization rate in the anticoagulant therapy group was increased (*P* < 0.00001) in patients with cirrhosis and portal vein thrombosis without increasing adverse events. Multiple use of enoxaparin in small doses is safer than single large doses (*P*=0.004). Direct oral anticoagulants are more effective (*P* < 0.00001) and safer than traditional anticoagulants. Prophylactic anticoagulant therapy can effectively prevent portal vein thrombosis formation (*P* < 0.00001).

**Conclusions:**

Anticoagulation therapy can treat or prevent portal vein thrombosis in patients with liver cirrhosis and is a relatively safe treatment.

## 1. Introduction

Portal vein thrombosis (PVT) is a common adverse event of liver cirrhosis, and its incidence increases as liver disease progresses and is even higher in patients with various portal hypertension procedures [1–3]. Patients with acute and severe PVT may experience symptoms, such as fever, abdominal pain, ascites, and splenomegaly, but many patients do not exhibit symptoms in the early stage of onset [4]. The hidden onset of PVT can cause significant harms to patients, including intestinal congestion and necrosis, secondary serious infections, increased risk of bleeding from esophageal varices rupture of the stomach, increased decompensation of the liver, more intraoperative and postoperative adverse events, and higher mortality [5, 6]. Therefore, to improve patient prognosis, timely and effective treatments of portal PVT are very important. As one of the main treatments of PVT, anticoagulation has received increasing attention in recent years, and prophylactic anticoagulation has even been proposed for patients at high risk of PVT. However, no definitive conclusion on anticoagulation effectiveness and safety has been reported. Some studies found that the recanalization rate of PVT after anticoagulation treatment is greater than 80% [7, 8]. However, other studies showed that anticoagulation treatment might be ineffective for PVT [9, 10]. Therefore, it is necessary to analyze relevant previous studies. This article is divided into two parts, namely, anticoagulation and prophylactic anticoagulation therapy, and both topics are analyzed using and meta-analysis to provide a reference for clinicians to treat or prevent PVT in patients with cirrhosis.

## 2. Materials and Methods

### 2.1. Document Retrieval

“Cirrhosis,” “liver cirrhosis,” “liver cirrhoses,” “hepatic cirrhosis,” “portal vein,” “thrombosis,” “thromboses,” “thrombus,” “blood clot,” “anticoagulant,” “anticoagulation,” “anticoagulant therapy,” “thrombin inhibitors” and other keywords were used to search databases, including PubMed, Embase, Cochrane Library, Web of Science, Wanfang, CNKI, and Weipu Database. The studies reported randomized controlled trials (RCT) and nonrandomized controlled trials (nRCT). No language limitations were imposed. This study included papers published up to December 2019.

### 2.2. Inclusion Criteria

① RCT or nRCT; ② study subjects were patients older than 18 years of age with liver cirrhosis at any stage attributed to various etiologies, and there were no restrictions on the race, nationality, or region; ③ the observation group was administered anticoagulants for anticoagulation, and the control group was treated with placebo or blank control, different anticoagulants, or different doses or treatment times with the same anticoagulant; ④ data reported should include these outcome indicators: portal vein recanalization or new onset, bleeding events, death, and other adverse events, including the new onset of decompensation of liver function, ascites, spontaneous peritonitis, sepsis, hepatorenal syndrome, or hepatic encephalopathy.

### 2.3. Exclusion Criteria

① Nonclinical research; ② studies for which a full text is not available; ③ republished literatures; ④ studies that do not provide complete data; ⑤ research subjects are noncirrhotic patients; ⑥ subjects have an underlying primary blood disease, membranous obstruction of the inferior vena cava, or preexisting extrahepatic thrombosis; ⑦ interventions other than anticoagulation; ⑧ research that is not germane to our subject.

### 2.4. Screening and Quality Evaluation

After reading the titles and abstracts of all the retrieved studies, preliminary screening was performed. The full text of the documents that passed the preliminary screening was read to exclude documents that clearly do not meet the requirements or are duplicate studies. The Cochrane bias risk assessment tool was used to assess the bias risk of included RCTs, and the Newcastle–Ottawa Scale (NOS) was used to assess the quality of included nRCTs.

### 2.5. Data Extraction and Statistical Analysis

Data extracted from each study included the following: first author, year of publication, country of publication, number of patients, liver function score, specific interventions, overall follow-up time, portal vein recanalization or new occurrence, bleeding events, other adverse events, and death.

## 3. Results

**Figure 1:**
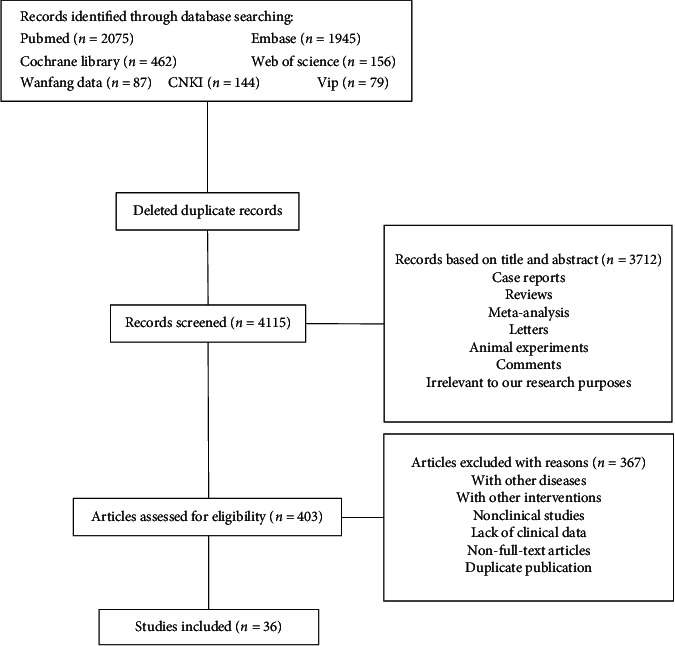
Flow chart of literature screening.

A total of 403 articles passed the preliminary screening, and 302 were excluded due to noncompliance of the study subjects or the use of intervention methods other than anticoagulation. In addition, 29 were nonclinical studies, and the full text of 16 articles could not be obtained. Moreover, 20 articles did not meet the requirements. Thus, thirty-six papers [2, 9, 11–44] were ultimately selected to complete this meta-analysis (Figure 1).

### 3.1. Basic Characteristics of Included Literatures

Of the 36 selected papers, 21 were reported in English, and 15 were in Chinese. Of the selected papers, 11 studies reported RCTs, and 25 reported nRCTs. The study sites included China, the United States, Europe, Japan, and other places. The dates of publication ranged from 2005 to 2019, and a total of 3479 patients were included. The basic characteristics of the included studies are provided in Table 1.

### 3.2. Bias Risk Assessment

**Figure 2:**
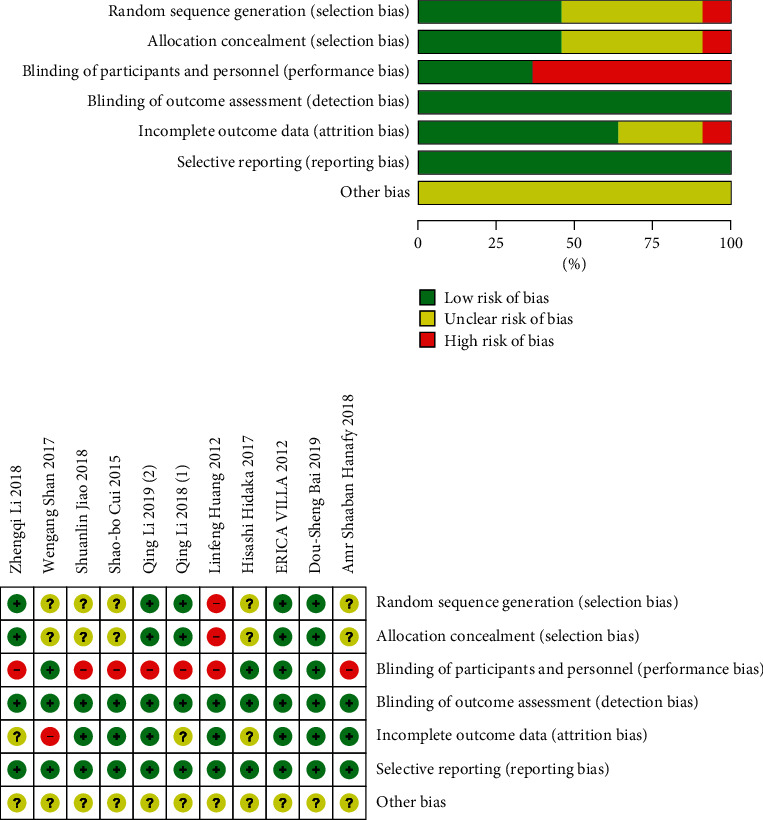
Bias analysis: (a) A review of the authors' judgments about each risk of bias item presented as percentages; (b) A review of the authors' judgments about each risk of bias item for included studies.

Cochrane bias risk assessment tool and NOS scale were selected for evaluation, as shown in Figures 2(a) and 2(b) and Table 2.

### 3.3. Statistical Results of Anticoagulant Therapy

**Figure 3:**
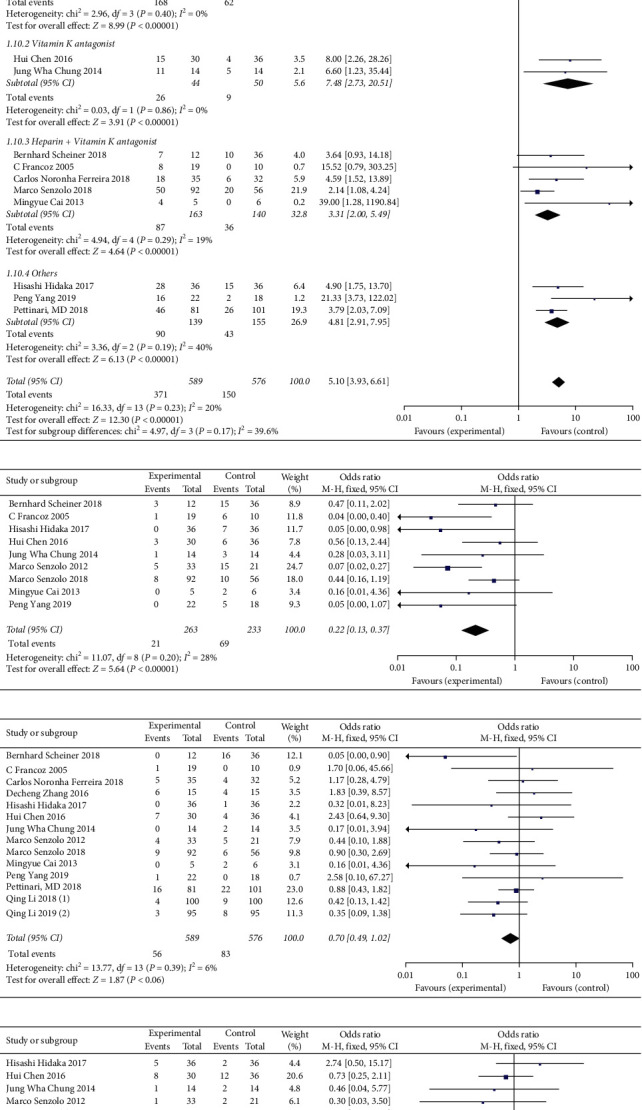
Effect and safety of anticoagulant therapy: (a) analysis of recanalization rate; (b) subgroup analysis of recanalization rate; (c) analysis of thrombus progression or rate of new thrombus formation; (d) bleeding events; (e) other adverse events; (f) mortality rate.

Figure 3(a) shows that the PVT recanalization rate in the observation group (anticoagulation) is increased compared with the control group, and the results are statistically significant (OR = 5.10, 95% CI: 3.93∼6.61, *P* < 0.00001). Subgroup analysis based on different drugs (other represents other anticoagulants, heparin, and/or warfarin combined with others) (Figure 3(b)) more specifically shows that different anticoagulants have therapeutic effects on PVT.  Figure 3 C shows that the thrombus progression or new thrombus formation in the observation group was reduced compared with the control group (OR = 0.22, 95% CI: 0.13∼0.37, *P* < 0.00001). Compared with the control group, anticoagulation did not increase the incidence of bleeding events (OR = 0.70, 95% CI: 0.49∼1.02, *P*=0.06) or the incidence of other adverse events (OR = 0.62, 95% CI: 0.37∼1.02, *P*=0.06), but the mortality rate was reduced (OR = 0.25, 95% CI: 0.08 ∼ 0.81, *P*=0.02) (Figures 2(d)–2(f)) (see Supplementary Figure 1 for histogram).

#### 3.3.1. Effect of Anticoagulant Therapy with Different Enoxaparin Doses

**Figure 4:**
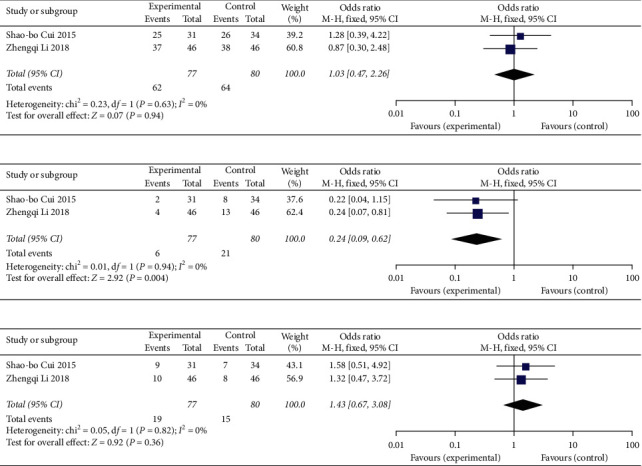
Effect and safety of anticoagulant with different doses of enoxaparin (1.0 mg/kg q 12 h in the experimental group and 1.5 mg/kg qd in the control group): (a) analysis of recanalization rate; (b) bleeding events; (c) other adverse events.

When different doses of enoxaparin were used for anticoagulation, the same effects were noted in the observation group (1.0 mg/kg q 12 h) and the control group (1.5 mg/kg q 24 h) of patients with liver cirrhosis and PVT (OR = 1.03, 95% CI: 0.47∼2.27, *P*=0.94) (Figure 4(a)), but the incidence of bleeding events was reduced in the former (OR = 0.24, 95% CI: 0.09∼0.62, *P*=0.004) (Figure 4(b)). No significant difference in the incidence of other adverse events was between the two groups (OR = 1.43, 95% CI: 0.67∼3.08, *P*=0.36) (Figure 4(c)) (see Supplementary Figure 2 for histogram).

#### 3.3.2. Therapeutic Effects of Direct Oral Anticoagulants (DOAC) vs. Traditional Anticoagulants

**Figure 5:**
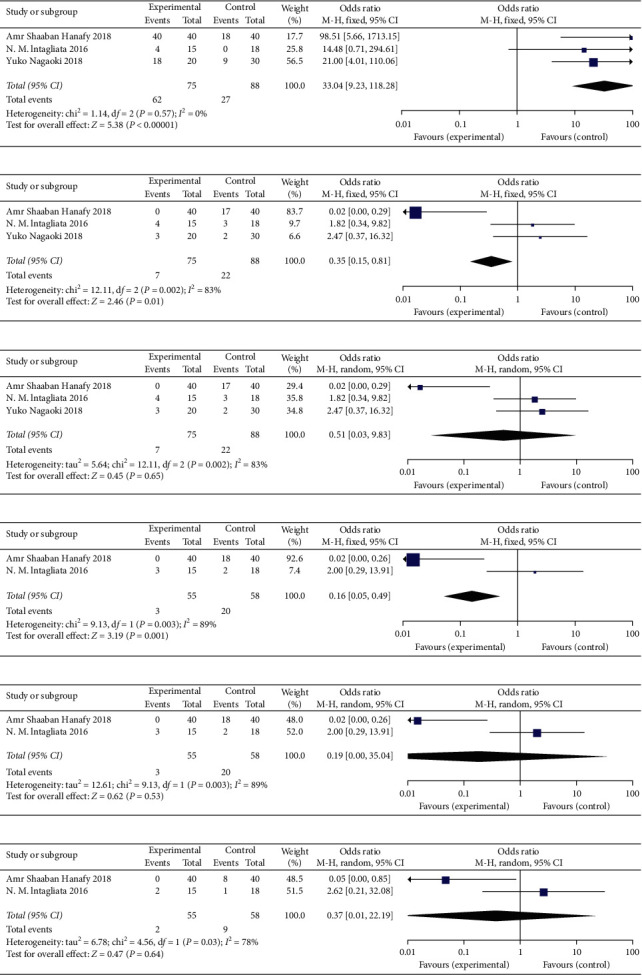
Therapeutic effects of direct oral anticoagulants vs. traditional anticoagulants (Experimental group: direct oral anticoagulant; Control group: traditional oral anticoagulant): (a) analysis of recanalization rate; (b) bleeding events; (c) analysis of bleeding events after random effects were combined; (d) other adverse events; (e) analysis of other adverse events after random effects were combined; (f) analysis of death events after random effects were combined.

The thrombus recanalization rate in the observation group (DOAC) was increased compared with the control group (traditional anticoagulant) (OR = 33.04, 95% CI: 9.23∼118.28, *P* < 0.00001) (Figure 5(a)). Apparently, bleeding (OR = 0.35, 95% CI: 0.15∼0.81, *P*=0.01) and other adverse events (OR = 0.16, 95% CI: 0.05∼0.49, *P*=0.001) in the observation group were reduced compared with the traditional anticoagulant group (Figures 5(b) and 5(d)). However, given the significant heterogeneity, the random effect model was used to merge the data. And the differences between the two groups were not statistically significant, including the incidence of bleeding events (OR = 0.51, 95% CI: 0.03∼9.83, *P*=0.65), risk of other adverse events (OR = 0.19, 95% CI: 0.00∼35.04, *P*=0.53), and death (OR = 0.37, 95% CI: 0.01∼22.19, *P*=0.64) (Figures 5(c), 5(e) and 5(f)) (see Supplementary Figure 3 for histogram).

### 3.4. Statistical Results of Prophylactic Anticoagulation

#### 3.4.1. Effect and Safety of Prophylactic Anticoagulation

**Figure 6:**
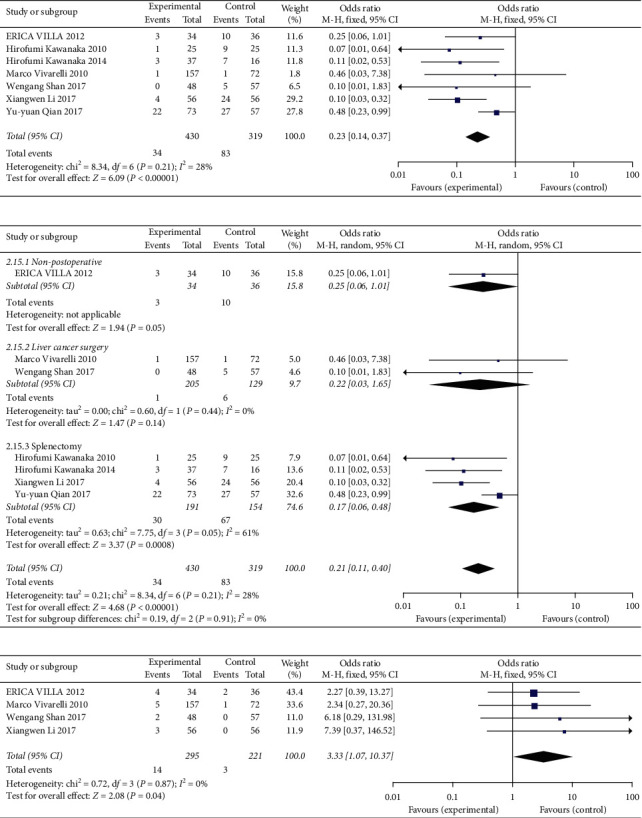
Effect and safety of prophylactic anticoagulant: (a) appearance of new thrombosis; (b) subgroup analysis of new thrombosis; (c) bleeding events.

The rate of PVT in the observation group (prophylactic anticoagulation treatment) was reduced compared with the control group, and the results were statistically significant (OR = 0.23, 95% CI: 0.14∼0.37, *P* < 0.00001) (Figure 6(a)). Using subgroup analysis, we found that the incidence of thrombosis in patients after splenectomy was significantly reduced compared with the control group (OR = 0.17, 95% CI: 0.06∼0.48, *P*=0.0008), but the difference was not significant in patients with liver cirrhosis after cancer resection (OR = 0.22, 95% CI: 0.03∼1.65, *P*=0.14) or no operation (OR = 0.25, 95% CI: 0.06∼1.01, *P*=0.05) (Figure 6(b)). The incidence of bleeding events in the observation group was increased compared with the control group (OR = 3.33, 95% CI: 1.07∼10.37, *P*=0.04) (Figure 6(c)) (see Supplementary Figure 4 for histogram).

#### 3.4.2. The Effect of Prophylactic Anticoagulation with Different Drugs

**Figure 7:**
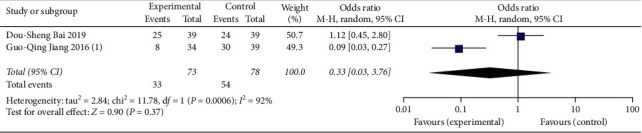
Effect of prophylactic anticoagulation with different drugs.

During preventive anticoagulation, the rate of thrombosis formation did not differ in the observation group (warfarin) and the control group (aspirin) (OR = 0.33, 95% CI: 0.03∼3.76, *P*=0.37) (Figure 7) (see Supplementary Figure 5 for histogram).

#### 3.4.3. Integration of Traditional Chinese and Western Medicine to Prevent PVT Formation

**Figure 8:**
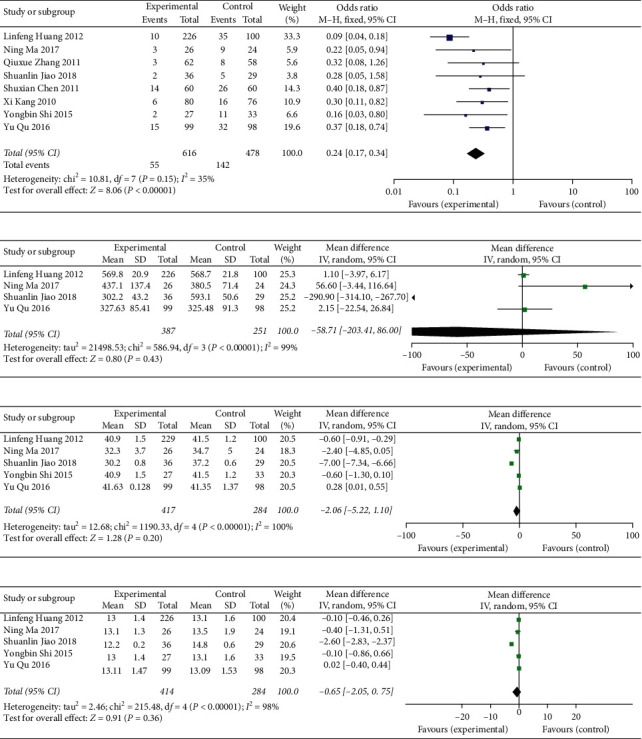
Effect and safety of anticoagulant combined with traditional Chinese medicine: (a) appearance of thrombosis and preventive effect of anticoagulant on PVT; (b) analysis of PLT; (c) analysis of APTT; (d) analysis of PT.

In anticoagulation therapies, the addition of drugs to promote blood circulation and prevent blood stasis can reduce the incidence of portal vein thrombosis (OR = 0.24, 95% CI: 0.17∼0.34, *P* < 0.00001) (Figure 8(a)). No significant differences in PLT (MD = −58.71, 95% CI: −203.41∼86.00, *P*=0.43), APTT (MD = −2.06, 95% CI: −5.22∼1.10, *P*=0.20), or PT (MD = −0.65, 95% CI: −2.05∼0.75, *P*=0.36) were noted between the two groups (Figures 8(b)–8(d)) (see Supplementary Figure 6 for histogram).

## 4. Discussion

The liver is an important organ that maintains the balance of the hemostatic system. As cirrhosis progresses, disorders of the coagulation and fibrinolytic system may occur, which can easily lead to bleeding and thromboembolism in patients. PVT, a serious adverse event of liver cirrhosis, is closely related to the hemodynamics of advanced portal hypertension. Its treatment methods include anticoagulation, thrombolysis, transjugular intrahepatic portal vein shunt (TIPS), and surgery. At present, few studies have assessed thrombolysis, and interventional therapy is generally suitable for patients with acute and severe PVT. Surgery is mainly used for patients with severe adverse events, such as uncontrollable gastrointestinal bleeding and intestinal necrosis caused by thrombosis. As a relatively noninvasive and simple treatment, anticoagulation represents one of the main clinical treatments for PVT. Anticoagulation therapy has achieved excellent results in the treatment of many cirrhosis patients with PVT and even patients with portal vein cavernous tumors [45–48]. However, the use of anticoagulants may cause some side effects, such as elevated liver enzymes, thrombocytopenia, prolonged prothrombin time, and even life-threatening cases [21, 49]. Therefore, the effectiveness and safety of anticoagulation therapy were further discussed in this article.

In terms of therapeutic anticoagulation, the results showed that anticoagulation prevents thrombus progression and increases the thrombosis recanalization rate. It is worth mentioning that in a study [50], the portal cavernomas were disappeared in two patients after anticoagulation. In addition, compared with the control group, bleeding events and other adverse events did not increase, and the mortality rate was decreased in the observation group. These results show that anticoagulation can treat cirrhosis PVT and improve patient survival without increasing side effects. Studies indicated that microthrombosis in the liver sinus exists in patients with cirrhosis [51, 52]. Microthrombi can increase portal pressure and cause intimal fibrosis and venous occlusion, eventually causing adjacent liver cells to be lost and replaced by fibrous tissue. Anticoagulation can improve liver fibrosis by combating microthrombosis, further improving liver function and reducing portal hypertension. Francoz et al. [12] found that liver function and renal function were improved in patients treated with enoxaparin. He also noted that enoxaparin could reduce intestinal cell damage by improving intestinal microcirculation, thereby reducing bacterial translocation. The Thrombosis Canada and 7th International Coagulation in Liver Disease Conference recommended liver transplant candidates with PVT for anticoagulation therapy and pointed out nontransplant candidates with acute PVT may also benefit [53]. Therefore, anticoagulation represents a safe, effective, and reliable option for patients with cirrhosis PVT, even those with poor liver function.

The 2016 Consensus of the Italian Society of Hepatology and the Italian Medical Association: Hemostasis Balance of Cirrhosis reported that thromboprophylaxis is not absolutely contraindicated in patients with cirrhosis [54]. However, through repeated searches of these literature libraries, only one controlled study [29] on preventive anticoagulation in nonsurgical cirrhosis patients was identified. Villa et al. found that enoxaparin was safe in preventing PVT in cirrhosis patients and delayed the occurrence of hepatic decompensation. However, related studies remain scarce. The possible reasons are as follows [8, 21, 55]: anticoagulation has serious side effects; PVT does not occur in all patients with cirrhosis; some PVT has a very high rate of spontaneous recanalization; and even if PVT is resolved with the use of anticoagulants, it may recur after stopping treatment. Many scholars have employed preventive anticoagulation after splenectomy or cancer resection in patients with liver cirrhosis. The surgical process and postoperative recovery may lead to a persistent hypercoagulable state, hemodynamic changes of the portal vein system, and local vascular disease, further promoting the occurrence of PVT [56, 57]. Our data shows that compared with the control group, PVT risk in the observation group does not decrease in patients with liver cirrhosis after cancer resection, but the risk did increase in patients after splenectomy, which is consistent with previous studies [58, 59]. However, whether preventive anticoagulation should be a routine treatment for patients with liver cirrhosis remains unclear because the study included in this article assessed patients after surgery for cirrhosis. Our data shows that the incidence of bleeding events in the observation group is higher than that in the control group. We believe that preventive anticoagulation is worth considering in those patients at high risk of PVT, such as those undergoing splenectomy.

Given that common anticoagulants have advantages and disadvantages, they should be used with the principle of “individualization.” Our results show that the effect of direct oral anticoagulants is improved compared with traditional anticoagulants, and warfarin and aspirin exhibit no significant differences when used in prophylactic anticoagulant therapy. In addition, the combination of traditional Chinese and Western medicine can also achieve good results without increasing the risk of abnormal blood clotting. Intagliata et al. [27] reported that dabigatran or rivaroxaban combined with antiplatelet agents is safer compared with warfarin. Despite these findings, we still need to choose the ideal drug based on the actual situation of the patient. The first factor to consider is pharmacokinetics, especially the functional state of the liver and kidney, which are involved in drug metabolism and clearance. A reduced glomerular filtration rate (GFR) will affect the pharmacokinetics of low molecular weight heparin (LMWH), and the low density of antithrombin-III in patients with liver cirrhosis may lead to heparin resistance [21]. Patients with renal insufficiency should avoid using dabigatran. The pharmacodynamics of rivaroxaban may be enhanced in patients with liver cirrhosis with poor liver function, while edoxaban, a new oral anticoagulant, is not metabolized by the liver [28, 60]. The interaction of drugs with food and other drugs cannot be ignored. For example, some foods rich in vitamin K and antibiotics and other drugs can affect the activity of CYP2C9 enzymes and potentially interfere with the efficacy of warfarin [61]. Economic capacity and compliance should also be taken into account. From our results, it seems that heparin is safer than vitamin K antagonists during the treatment of PVT. However, the high cost, preservation conditions, and daily injection of LMWH cause medical centers to prefer vitamin K antagonists [62]. For emergency operations, the effect of LMWH exhibits a shorter duration, and the dosage can be adjusted easily and accurately. Thus, LMWH is better than VKA [12]. The Consensus Statement of the 7th Meeting on Coagulation of Liver Disease suggests that it is important to use direct oral anticoagulants (DOACs) as a treatment option for compensatory liver cirrhosis. LMWH is preferred in an emergency, and treatment should continue until hepatic decompensation is stable. In addition, long-term anticoagulation DOACs can be considered as a safe alternative. DOACs are an effective choice for anticoagulant therapy for patients with heparin-induced thrombocytopenia [15].

Next, we should clarify specific treatment dosages and anticoagulant regimens. In the studies included in this article, the dose and timing of anticoagulant drugs are subjective, and currently, no international standard exists for these parameters. Only two articles discussed the use of enoxaparin and found that it is safer to use it in small doses and at multiple times. The anticoagulant time suggested in each guideline or consensus also varies. The American Association for the Study of Liver Diseases (AASLD) recommends anticoagulant therapy for at least 3 months to recanalize the PVT in cases with the deterioration of intestinal infarction and portal hypertension [63]. In 2018, the National Comprehensive Cancer Network (NCCN) recommended anticoagulation for at least 6 months without contraindications [64]. In patients with superior mesenteric vein thrombosis, with a past history suggestive of intestinal ischemia or liver transplant candidates, the European Association for the Study of Liver recommended lifelong anticoagulation [65]. The clinical evidence for these problems is inadequate, and data from more clinical trials are needed to support these findings.

In addition, the effects of anticoagulant therapy are affected by many factors, such as age, liver function score, thrombus condition, platelet count, time of thrombosis, hepatic encephalopathy, and hereditary thrombotic disease [13, 16]. Delgado et al. [55] proposed that anticoagulant therapy should begin as early as 2 weeks before the discovery of thrombosis because the processes of fibrosis in chronic PVT are irreversible. One study reported that SMV thrombus is an important parameter related to the continuous recanalization of the portal vein. When the PVT extends out of the SMV and the flow rate is reduced by 50%, the anticoagulant effect may be offset by a reduced flow rate [66]. Varicose veins rupture, so bleeding is also associated with PVT recanalization [13]. The 2015 European Guidelines for Hepatic Vascular Disease state that it is important to fully assess the risk of acute bleeding or esophageal and gastric variceal rupture bleeding prior to anticoagulant therapy and to prepare methods to prevent bleeding [65]. It should be noted that approximately 70% to 75% of PVTs occur in malignant tumors [67]. The prognosis of patients with tumor thrombus infiltration is extremely poor, so the use of anticoagulants is not recommended. Therefore, attention should also be paid to distinguish a cancer thrombus from a benign thrombus by the combined judgment of imaging features and alpha-fetoprotein levels before anticoagulant treatment [66]. In summary, the clinical decision-making process for anticoagulant therapy requires many comprehensive considerations.

A major limitation of this study is that some articles are nonrandomized controlled trials. These studies carry a certain level of bias, such as patient selection, drug dosage and course, treatment evaluation, and follow-up. In addition, the lack of patients stratification according to the severity of cirrhosis (compensated/decopensated, CP class A/B/C, MELD, etc.…) in the evaluation of treatment effects prevents us from determining whether all patients with cirrhosis should be treated with anticoagulation. Anticoagulant therapy based on combined traditional Chinese and Western medicine seeks to promote blood circulation by preventing blood stasis during PVT treatment. Preventive anticoagulation also requires comparative clinical trials between the anticoagulant with and without traditional Chinese medicine to further confirm the effect on promoting blood circulation and preventing blood stasis. The longest median follow-up time in the study in this paper is 5 years, and the effect of anticoagulants on long-term prognosis requires further study.

## 5. Summary

PVT is a serious adverse event in patients with cirrhosis. The results show that anticoagulant therapy can effectively and safely treat PVT in patients with cirrhosis and effectively reduce the mortality rate. In addition, this paper also demonstrates that prophylactic anticoagulant therapy can prevent PVT after splenectomy. The necessity of prophylactic anticoagulant therapy requires further discussion. In cases without contraindications, anticoagulants are recommended for liver cirrhosis patients with PVT. The selection of anticoagulant drugs and the dosage and course of drugs should be considered based on the patient's conditions.

## Figures and Tables

**Table 1:** Basic characteristics of included studies.

Author	Year	Country	Journal	Type of study	Research objects	Follow-up time	Anticoagulation	Number of study	Gender (male/female)	Age
Anticoagulant therapy:										
Scheiner et al. [11]	2018	Austria	Wien Klin Wochenschr	Retrospective	Liver cirrhosis patients with PVT	44.1 months	Low molecular weight heparin (LMWH) or warfarin	Observation group 12	32/19	52.9 ± 12.5
								Control group 39		
Francoz et al. [12]	2005	France	Gut	Prospective	Liver cirrhosis patients with PVT	7.9 ± 6.2 months	LMWH + vitamin K antagonists (VKA)	Observation group 19	13/6	48.7 ± 7.5
						5.8 ± 4.6 months		Control group 10	7/3	52 ± 5.7
Noronha Ferreira et al. [13]	2018	Portuguese	Digestive diseases and Sciences	Retrospective	Liver cirrhosis patients with PVT	25.5 months (1–146)	LMWH or warfarin	Observation group 37	20/17	59 ± 8
								Control group 43	25/18	60 ± 10
Zhang [14]	2016	China	Graduation Thesis of Anhui Medical University	Retrospective	Liver cirrhosis patients with PVT	12 months	LMWH	Observation group 15	—	—
								Control group 15	—	—
Hidaka et al. [15]	2017	Japan	Hepatology research	Prospective	Liver cirrhosis patients with PVT	After each treatment period 16 (±3) days	Antithrombin III thrombin-antithrombin complex (TAT)	Observation group 36	26/10	66 (39 − 80)
								Control group 36	20/16	69.5 (48 − 86)
Chen et al. [16]	2016	China	Wolters Kluwer Health	Retrospective	Liver cirrhosis patients with PVT	33.2 ± 29.2 months	Warfarin	Observation group 30	23/7	44.97 ± 12.3
						25.9 ± 23 months		Control group 36	24/12	47.86 ± 10.6
Chung et al. [17]	2014	Korea	Clinical and molecular Hepatology	Prospective	Liver cirrhosis patients with PVT	12 months	Warfarin	Observation group 14	10/4	59.4 ± 12
								Control group 14	11/3	58.7 ± 13.2
Senzolo et al. [9]	2012	Italy	Liver international	Prospective	Liver cirrhosis patients with PVT	22.53 ± 8.5 months	Nadroparin	Observation group 33	25/10	55.5 ± 5
		England						Control group 21	25/10	52.3 ± 4
Senzolo et al. [18]	2018	Italy	Clinical and translational gastroenterology	Prospective	Liver cirrhosis patients with PVT	6.5 months	Heparin, LMWH or Fondaparinux + VKA	Observation group 92	64/28	61 (52 − 69)
								Control group 56	42/14	56 (49 − 65.5)
Cai et al. [19]	2013	China	Journal of vascular and interventional radiology	Prospective	Liver cirrhosis patients with PVT	37.6 months	LMWH or warfarin	Observation group 5	10/1	52.8 (40 − 69)
								Control group 6		
Yang [20]	2019	China	Graduation thesis of Shanxi medical university	Prospective	Liver cirrhosis patients with PVT	6 months	LMWH/enoxaparin + warfarin, rivaroxaban, dabigatran	Observation group 22	—	—
								Control group 18	—	—
Pettinari et al. [21]	2018	Italy	The American College of gastroenterology	Retrospective	Liver cirrhosis patients with PVT	19 (3–94) months	LMWH, Sulfonated heparin, direct oral anticoagulants	Observation group 81	56/25	57.9 ± 11.1
								Control group 101	74/37	57.7 ± 11.3
Li et al. [22]	2018	China	China medical Herald	Prospective	Liver cirrhosis patients with PVT	6 months	LMWH	Observation group 100	62/38	50.5 ± 8.6
								Control group 100	60/40	52.7 ± 7.9
Li et al. [23]	2019	China	Modern digestion & intervention	Prospective	Liver cirrhosis patients with PVT	6 months	LMWH	Observation group 95	57/38	50.64 ± 8.33
								Control group 95	56/39	52.43 ± 7.15

Enoxaparin of different doses:										
Cui et al. [24]	2015	China	Wolters Kluwer Health	Prospective	Hepatitis b liver cirrhosis patients with acute PTV	6 months	Enoxaparin 1 mg/kg q12 h	Observation group 31	19/12	52.3 ± 10.1
							Enoxaparin 1.5 mg/kg qd	Control group 34	24/10	53.1 ± 10.1
Li [25]	2018	China	Chinese journal of integrated traditional and Western medicine on liver disease	Prospective	Liver cirrhosis patients with PVT	Up to 36 months	Enoxaparin 1 mg/kg q12 h	Observation group 46		
							Enoxaparin 1.5 mg/kg qd	Control group 46		

Anticoagulants therapy of different drugs (direct oral anticoagulants vs conventional anticoagulants):										
Hanafy et al. [26]	2018	Egypt	Vascular pharmacology	Prospective	Hepatitis C cirrhosis patients with PVT	12 months	Rivaroxaban	Observation group 40	35/5	41.3 ± 2.3
							Warfarin	Control group 40	32/8	46 ± 5
Intagliata et al. [27]	2015	America	Digestive diseases and Sciences	Retrospective	Liver cirrhosis patients with PVT	Up to 36 months	Apixaban rivaroxaban	Observation group 20	10/10	57 (50 − 64)
							Enoxaparin warfarin	Control group 19	12/7	60 (55 − 64)
Nagaoki et al. [28]	2018	Japan	Hepatol Res	Retrospective	Liver cirrhosis patients with PVT	6 months	Edoxaban	Observation group 20	13/7.0	69 (53 − 74)
							Warfarin	Control group 30	17/13	67 (24 − 83)

Prophylactic anticoagulant therapy:										
Villa et al. [29]	2012	Italy	Gastroenterology	Prospective	Liver cirrhosis patients	89 ± 57 weeks	Enoxaparin	Observation group 34	25/9	56 ± 5
						58 ± 37 weeks		Control group 36	26/10	57 ± 7
Kawanaka et al. [30]	2010	Japan	Annals of surgery	Prospective	Patients with liver cirrhosis who underwent splenectomy	3 months	Antithrombin III	Observation group 25	10/15	61 (45 − 76)
								Control group 25	16/9	56 (43 − 71)
Kawanaka et al. [31]	2014	Japan	American College of Surgeons	Prospective	Patients with liver cirrhosis who underwent splenectomy	3 months	Antithrombin III, LMWH, warfarin	Observation group 37	16/21	61.9 ± 8.8
								Control group 16	10/6	59.6 ± 8.3
Vivarelli et al. [32]	2010	Italy	World J gastroenterol	Retrospective	Patients with cirrhosis and liver cancer who underwent operation	12 months	Enoxaparin	Observation group 157	119/38	65 ± 9.8
								Control group 72	52/26	63 ± 9.5
Shan et al. [33]	2017	China	Acta Universitatis Medicinalis Nanjing	Prospective	Patients with liver cancer who underwent operation	1 week	LMWH	Observation group 48	38/10	58.71 ± 8.6
								Control group 57	45/12	56.79 ± 10.9
Li and Tu [34]	2017	China	Journal of Practical Hepatology	Prospective	Patients with liver cirrhosis who underwent splenectomy	2 weeks	LMWH	Observation group 56	71/41	46.8 ± 4.3
								Control group 56		
Qian and Li [2]	2017	China	International Journal of Surgery	Retrospective	Patients with liver cirrhosis who underwent splenectomy	1 month	LMWH, aspirin, warfarin	Observation group 73	38/35	72.2 ± 7.6
								Control group 57	31/26	72.3 ± 8

Prophylactic anticoagulant therapy with different drugs (warfarin vs aspirin)										
Bai et al. [35]	2019	China	International Journal of Surgery	Prospective	Patients with liver cirrhosis who underwent splenectomy	24 months	Warfarin	Observation group 39	24/15	52.2 ± 10.4
							Aspirin	Control group 39	27/12	50.5 ± 8.3
Jiang et al. [36]	2016	China	Journal of Laparoendoscopic & Advanced Surgical Techniques	Retrospective	Patients with liver cirrhosis who underwent splenectomy	3 months	Warfarin	Observation group 34	13/21.0	55.2 ± 10.3
							Aspirin	Control group 39	20/19.0	51.9 ± 8.7

Integration of traditional Chinese and Western medicine to prevent PVT:										
Huang et al. [37]	2012	China	China medical Herald	Prospective	Patients with liver cirrhosis who underwent splenectomy	3 months	Salviae miltiorrhizae radix/Danhong, aspirin, dipyridamole and LMWH	Observation group 226	147/79	45.87 + 8.46
								Control group 100	65/35	46.98 + 8.38
Ning [38]	2017	China	Graduation thesis of Jilin university	Retrospective	Patients with liver cirrhosis who underwent splenectomy	3–12 months	Ligustrazine, aspirin, LMWH	Observation group 26	18/8	48.0 ± 12.4
								Control group 24	16/8	53.4 ± 7.8
Zhang et al. [39]	2011	China	Hebei medicine	Prospective	Patients with liver cirrhosis who underwent splenectomy	3 months	Ligustrazine, aspirin, LMWH	Observation group 62	46/16	21 − 65
								Control group 58	47/11	23 − 62
Jiao et al. [40]	2018	China	Chinese Hepatology	Prospective	Patients with liver cirrhosis who underwent splenectomy	2 weeks	Salviae miltiorrhizae radix and LMWH	Observation group 36	26/10	45.84 ± 2.92
								Control group 29	20/9	44.96 ± 2.18
Chen et al. [41]	2011	China	China medical Herald	Retrospective	Patients with liver cirrhosis who underwent splenectomy	2 weeks	Ligustrazine or Salviae miltiorrhizae radix, dipyridamol/Aspirin, LMWH	Observation group 60	36/24	45.51 ± 13.28
								Control group 60	40/20	44.57 ± 13.56
Kang et al. [42]	2010	China	Clinical medicine practice	Prospective	Patients with liver cirrhosis who underwent splenectomy	3 months	Salviae miltiorrhizae radix and LMWH	Observation group 80	42/3447/33	44.16 ± 9.5745.87 ± 8.86
								Control group 76		
Shi et al. [43]	2015	China	Chinese journal of integrative medicine on Cardio/Cerebrovascular disease	Retrospective	Patients with liver cirrhosis who underwent splenectomy	2 months	Traditional Chinese medicine, aspirin, LMWH	Observation group 27		
								Control group 33		
Qu [44]	2016	China	International journal of geriatrics	Retrospective	Patients with liver cirrhosis who underwent splenectomy	3 months	Danhong, LMWH	Observation group 99	74/25	46.53 ± 3.14
								Control group 98	75/23	47.03 ± 2.98

**Table 2:** The quality of studies with NOS scores.

Studies	Selection	Comparability	Outcome	Stars
Scheiner et al. 2018 [11]	4	1	2	7
Francoz et al. 2005 [12]	4	2	3	9
Noronha Ferreira et al. 2018 [13]	4	2	3	9
Zhang 2016 [14]	4	0	3	7
Chen et al. 2016 [16]	4	2	3	9
Chung et al. 2014 [17]	4	2	3	9
Senzolo et al. 2012 [9]	3	2	2	7
Senzolo et al. 2018 [18]	4	2	2	8
Cai et al. 2013 [19]	3	0	3	6
Yang 2019 [20]	4	0	3	7
Pettinari et al. 2018 [21]	4	1	3	8
Intagliata et al. 2016 [27]	4	2	3	9
Nagaoki et al. 2018 [28]	4	2	3	9
Kawanaka et al. 2010 [30]	3	3	3	8
Kawanaka et al. 2014 [31]	4	1	3	8
Vivarelli et al. 2010 [32]	4	1	3	8
Li and Tu 2017 [34]	4	1	2	7
Harding et al. [6]	4	1	3	8
Jiang et al. 2016 [36]	4	2	3	9
Ning 2017 [38]	4	2	3	9
Zhang et al. 2011 [39]	4	0	3	7
Chen et al. 2011 [41]	4	0	3	7
Kang and Zhang 2010 [42]	4	1	3	8
Shi et al. 2015 [43]	4	1	3	8
Qu 2016 [44]	4	2	3	9

## Data Availability

The data used to support the findings of this study are included within the article.

## Supplementary Materials

Supplementary Materials Supplementary Figure 1: histogram of effect and safety of anticoagulant therapy. Supplementary Figure 2: histogram of effect and safety of anticoagulant with different doses of enoxaparin. Supplementary Figure 3: histogram of effect and safety of direct oral anticoagulants vs. traditional anticoagulants. Supplementary Figure 4: histogram of effect and safety of preventive anticoagulant. Supplementary Figure 5: histogram of effect and safety of prophylactic anticoagulation with different drugs. Supplementary Figure 6: histogram of effect and safety of anticoagulant combined with traditional Chinese medicine.
