# Supplementary material for: Safety and Efficacy of Anticoagulation in Patients with Cirrhosis: A Meta-Analysis
Source: Can J Gastroenterol Hepatol. 2021 Apr 21;2021:8859602. doi: 10.1155/2021/8859602 (PMC8102101; doi:10.1155/2021/8859602)
Supplement: Supplementary Materials — Supplementary Figure 1: histogram of effect and safety of anticoagulant therapy. Supplementary Figure 2: histogram of effect and safety of anticoagulant with different doses of enoxaparin. Supplementary Figure 3: histogram of effect and safety of direct oral anticoagulants vs. traditional anticoagulants. Supplementary Figure 4: histogram of effect and safety of preventive anticoagulant. Supplementary Figure 5: histogram of effect and safety of prophylactic anticoagulation with different drugs. Supplementary Figure 6: histogram of effect and safety of anticoagulant combined with traditional Chinese medicine. [file 8859602.f1.docx]

Supplementary figure 1





Supplementary figure 2





Supplementary figure 3





Supplementary figure 4





Supplementary figure 5





Supplementary figure 6
